# The Effects of *Enterococcus faecium* and Selenium on Methotrexate Treatment in Rat Adjuvant-induced Arthritis

**DOI:** 10.1080/17402520400001660

**Published:** 2004

**Authors:** Jozef Rovenský, Karol Švík, Vladimír Maťha, Richard Ištok, Ladislav Ebringer, Miroslav Ferenčík, Mária Stančíková

**Affiliations:** 1 National Institute of Rheumatic Diseases Piešt'any Slovakia; 2 Institute of Cell Biology Faculty of Science Comenius University Bratislava Slovakia; 3 Institute of Immunology Faculty of Medicine Comenius University Bratislava Slovakia

## Abstract

The effects of probiotic bacteria *Enterococcus faecium* (EF) and selenium were studied on methotrexate (MTX) treatment in rats with adjuvant arthritis (AA).

Arthritic rats were preventive treated orally with the following substances: lyophilized EF (15 mg/kg/day, 5 days a week); sodium selenite pentahydrate (SSe, 0.050 mg/kg containing 0.015 mg/kg selenium, 5 days a week); MTX (0.6 mg/kg/week), and their combinations for the period of 50 days from adjuvant application. Levels of serum albumin, serum nitrite/nitrate concentrations, hind paw swelling, arthrogram scores, whole body bone mineral density (BMD), and bone erosions were evaluated as markers of inflammation and destructive changes associated with arthritis.

Long-term preventive treatment with low-dose MTX significantly inhibited the markers of both inflammation and arthritis. EF or SSe when administered singly or in combination had no significant effect on given parameters in arthritic rats. EF but not SSe potentiated the beneficial effects of MTX, which resulted in a more significant reduction of hind paw swelling, arthrogram scores and whole body BMD decrease. EF had a tendency to improve also the effect of MTX on serum albumin and nitrite/nitrate concentrations.

Our results indicate that EF may increase the preventive effect of MTX treatment in rat AA by improving its anti-inflammatory and anti-arthritic effects.


*Clinical & Developmental Immunology*, September/December 2004, Vol. 11 (3/4), pp. 267–273

Taylor & Francis healthsciences

### The Effects of *Enterococcus faecium* and Selenium on Methotrexate Treatment in Rat Adjuvant-induced Arthritis

JOZEF ROVENSKÝ[a,*], KAROL ŠVÍK[a], VLADIMÍR MAŤHA[a], RICHARD IŠTOK[a], LADISLAV EBRINGER[b], MIROSLAV FERENČÍK[c] and MÁRIA STANČÍKOVÁ[a]

[a]*National Institute of Rheumatic Diseases, Nabrezie I. Krasku 4, 921 12 Piešt'any, Slovak Republic;* [b]*Institute of Cell Biology, Faculty of Science, Comenius University, 811 07 Bratislava, Slovak Republic;* [c]*Institute of Immunology, Faculty of Medicine, Comenius University, 811 08 Bratislava, Slovak Republic*

The effects of probiotic bacteria *Enterococcus faecium* (EF) and selenium were studied on methotrexate (MTX) treatment in rats with adjuvant arthritis (AA).

Arthritic rats were preventive treated orally with the following substances: lyophilized EF (15 mg/kg/day, 5 days a week); sodium selenite pentahydrate (SSe, 0.050 mg/kg containing 0.015 mg/kg selenium, 5 days a week); MTX (0.6 mg/kg/week), and their combinations for the period of 50 days from adjuvant application. Levels of serum albumin, serum nitrite/nitrate concentrations, hind paw swelling, arthrogram scores, whole body bone mineral density (BMD), and bone erosions were evaluated as markers of inflammation and destructive changes associated with arthritis.

Long-term preventive treatment with low-dose MTX significantly inhibited the markers of both inflammation and arthritis. EF or SSe when administered singly or in combination had no significant effect on given parameters in arthritic rats. EF but not SSe potentiated the beneficial effects of MTX, which resulted in a more significant reduction of hind paw swelling, arthrogram scores and whole body BMD decrease. EF had a tendency to improve also the effect of MTX on serum albumin and nitrite/nitrate concentrations.

Our results indicate that EF may increase the preventive effect of MTX treatment in rat AA by improving its anti-inflammatory and anti-arthritic effects.

*Keywords*: Adjuvant arthritis; *Enterococcus faecium*; Methotrexate; Selenium

### INTRODUCTION

Increasing interest in an influence of intestinal microflora on human and animal health has resulted in attempts to improve optimally its composition by using probiotics. Probiotics are defined as live cultures of microorganisms that, if administered in sufficient quantities, beneficially affect the host by improving its intestinal microbial balance (Reid *et al.*, 2003). They influence favourably both development and stability of the microflora, inhibit colonization by pathogens, influence the mucosal barrier by their trophic effect on intestinal epithelium and stimulate both specific and non-specific components of the immune system (Isolauri *et al.*, 2001; Tlaskalová-Hogenová *et al.*, 2004). The bacteria most frequently used as probiotics are those from *Lactobacillus* and *Bifidobacter* strains. However, some non-pathogenic strains of *E. coli* proved to be suitable for this purpose as well.

*Enterococcus faecium* (EF), like many other lactic-acid bacteria in functional foods, can transiently colonize the human intestine and exert beneficial probiotic effect (Belicová *et al.*, 1999). Significant immunostimulatory effects on both phagocytosis by neutrophils and antibody production by EF have been described in several studies (Mikeš *et al.*, 1995; Ferenčík *et al.*, 2000). As a food additive, this probiotic agent is available also in the selenium-enriched form. Selenium is an essential trace element with antioxidant properties able to modulate the anti-inflammatory and immune responses (Rayman, 2000). Deficiencies in selenium attenuate especially the cellular immune response by oxidative stress, and increase the risk of bacterial and viral infections. Inflammatory and arthritic manifestations of adjuvant arthritis (AA) in rats significantly worsened after 6 and 12 weeks of application of selenium-deficient diet (Parnham *et al.*, 1983). Selenium supplementation in RA patients did not decrease the arthritic score but improved some symptoms of the disease (Peretz *et al.*, 2001). The combination of EF with organic selenium may enhance the immunostimulatory and anti-inflammatory actions of this probiotic agent.

*Corresponding author. Address: Nábrežie I. Krasku 4, 921 12 Piešťany, Slovak Republic. Tel.: +42-133-7723586. Fax: +42-133-7721192. E-mail: rovensky@nurch.sk

ISSN 1740-2522 print/ISSN 1740-2530 online © 2004 Taylor & Francis Ltd
DOI: 10.1080/17402520400001660

Long lasting unfavourable composition of intestinal microflora could be associated with occurrence of diseases that may have no relation to the bacteria living in intestines; it may also increase susceptibility to infectious diseases or worsen absorption of some drugs. Certain association was observed among gastrointestinal system, arthritis and immune system (Sartor, 1997; Cebra, 1999). Patients with newly diagnosed RA have their intestinal microflora composition altered (especially in the case of Gram-positive anaerobic bacteria) in comparison with healthy controls (Eerola *et al.*, 1994). It has also been suggested that in arthritis the intestinal defensive barrier is disturbed (Malin *et al.*, 1997). Short-term therapy with *Lactobacillus GG* showed a tendency to increase the IgA secretion and thus improve the mucosal barrier mechanism (Hatakka *et al.*, 2003). The positive alterations in intestine flora can be induced also by uncooked, lactobacilli-rich, vegan diet. Decrease of RA activity (Peltonen *et al.*, 1997) or the subjective symptoms of the disease (McDougall *et al.*, 2002) were observed in RA patients using the vegan diet.

Kano *et al.* (2002) have shown that oral intake of *Lactobacillus delbrueckii* subsp. *bulgaricus* OLL1073R-1 prevents development of collagen-induced arthritis in mice. On the other hand it has been suggested that the Gram-positive bacteria belonging to the normal intestinal flora (e.g. *Eubacterium aerofaciens*, *Lactobacillus casei*) may induce arthritis and the production of pro-inflammatory cytokines in rats (Chen *et al.*, 1999; Simelyte *et al.*, 2000). The beneficial and the harmful effects of probiotics are clearly strain-dependent, therefore it is important to test different probiotics and their affect on the development and course of arthritis. This work is continuation of our previous study with selenium-enriched EF (Rovensky *et al.*, 2002). The results of our previous study proved that this probiotic agent had a tendency to improve some inflammatory and arthritic parameters in AA rats and significantly potentiate the preventive effect of methotrexate (MTX). MTX is widely used in RA therapy for its antiinflammatory and immunosuppressive effects. However, one of its side effects is the damage of intestinal mucosa.

The purpose of the present study is to answer the question whether the beneficial effect of probiotic agent selenium-enriched EF on the MTX treatment is a result of the single EF, or selenium, or both of these components.

### METHODS

### Materials

MTX and sodium selenite pentahydrate (SSe) were purchased as pure substances from Pliva-Lachema Ltd. (Brno, Czech Republic). EF M74—the lyophilized probiotic agent containing $360 \times 10^9/\mathrm{g}$ CFU (colony forming units) was prepared by MEDIPHARM (Hustopeče, Czech Republic). *Mycobacterium butyricum* in lyophilized form was purchased from Difco Laboratories Co. Ltd. (Detroit, USA), and the incomplete Freund's adjuvant was purchased from Sigma-Aldrich Chemie GmbH (Taufkirchen, Germany).

#### *Animals*

Male Lewis rats (170 ± 10 g; Charles River Wiga, Sulzfeld, Germany) were maintained during the experiment in standard animal facilities that comply with the European Convention for the Protection of Vertebrae Animals Used for Experimental and Other Scientific Purposes. The animals were fed pelleted food (TOP DOVO, Dobrá Voda, Slovak Republic) and had free access to both food and water. The State Veterinary and Food Committee of the Slovak Republic and the Ethics Committee for Control of Animals Experimentation on the National Institute of Rheumatic Diseases approved the experimental protocol and all procedures.

#### *Induction of Arthritis*

The rats were injected with 0.1 ml suspension of heat-killed *M. butyricum* (12 mg/ml) in incomplete Freund's adjuvant intradermally at the base of the tail.

### Treatment

Tested substances were administered in corresponding doses from day 0 (day of immunization) to day 50 of the study. Lyophilized EF (15 mg/kg), SSe (0.050 mg/kg containing 0.015 mg/kg selenium), and their combinations were prepared in distilled water to reach the required concentration and were applied *per os* in once-daily dose, 5-times a week. MTX was prepared in sterile saline to yield the desired concentration of 0.3 mg/kg in 0.1 ml saline, and applied twice a week *per os* (0.6 mg/kg in total per week). A fresh solution of the tested substances was prepared on each day of the administration. The untreated groups received vehicle (sterile saline) in the same manner daily for 50 days.

#### *Rat Groups*

The animals were divided into the following 9 groups of ten: Group 1: non-arthritic untreated controls; Group 2: untreated rats with AA; Group 3: AA rats treated with EF; Group 4: AA rats receiving SSe; Group 5: AA rats administered the combination of EF + SSe; Group 6: AA rats treated with MTX; Group 7: AA rats treated with the combination of MTX + EF; Group 8: AA rats treated with the combination of MTX + SSe; Group 9: AA rats administered the combination of MTX + EF + SSe.

### Evaluated Parameters

#### *Hind Paw Swelling*

The volume of the hind paws swelling was measured with an electronic water plethysmometer (UGO BASILE, Comerio-Varese, Italy) on days 14, 21 and 28.

### *Arthrogram Score*

The severity of arthritis was quantified by scoring each paw from 1 to 5, based on increasing levels of swelling and periarticular erythema. The sum of the scores for the limbs was calculated as the arthritic index, with a maximum possible score of 20 per rat. Arthrogram scores were evaluated on days 14, 21 and 28.

### *Serum Albumin Levels*

Serum albumin levels were measured on days 14, 21 and 28 in the rat serum by spectrophotometric method, using SYS 1 kit (BM/Hitachi, Boehringer Mannheim, Germany) on a Hitachi 911 automatic biochemical analyzer.

### *Serum Nitrite/nitrate*

Nitrite/nitrate concentration in deproteinised serum was determined by the method of Cortas and Waking (1990) on days 14, 21 and 28. Cu-coated cadmium granules in glycine buffer at pH 9.7 reduced nitrate and the resulting nitrite was evaluated by Griess reaction.

### *Bone Mineral Density (BMD)*

BMD was measured by dual-energy X-ray absorptiometry (DEXA) using a Hologic QDR®-4500 (Waldham, HA, USA) with the equipment for measuring small laboratory animals. The whole body BMD of rats was determined on day 50 after immunization.

### *Bone Erosions*

Bone changes, indicated by erosions of tarsal and metatarsal bone structures of hind paws, were evaluated from radiographic prints. They were taken on a computer controlled X-ray generator (Philips Super 80CP, Hamburg, Germany) on day 50 after immunization, using the modified arthrogram scoring system (Welles *et al.*, 1985). Every hind paw radiograph was checked for matching to one of the 5 degrees of bone erosion: degree 1: osteoporosis of the distal part of the tibia and the tarsal bones; degree 2: hyperostosis with osteophytes in the tibiotarsal joint region; degree 3: as in degree 2 plus hyperostosis with osteophytes in the tarsometatarsal joint region; degree 4: as in degree 3 plus hyperostosis with osteophytes in the tarsometatarsalophalangeal joint region; degree 5: as in degree 4 plus deformation of the joint spaces.

### Statistical Analysis of the Results

One-way analysis of variance (ANOVA) was used for statistical analysis of the results and $p < 0.05$ was taken as the significance limit for all comparisons.

### RESULTS

### Hind Paw Swelling

The hind paw swelling reflects both inflammatory and arthritic changes occurring in rats with AA. The volume of the swollen hind paws in arthritic rats on day 21 was about twice of that found in healthy controls (Table I). The statistically significant decrease was observed in rats treated with MTX compared to untreated arthritic controls. The reduction of hind paw volume was more pronounced in rats treated with the combination of MTX + EF than with MTX alone ($p < 0.05$). Selenium alone had no effect on MTX treatment. The best preventive effect observed with the 3-agent combination of MTX + EF + SSe may be also the result of the presence of EF in this treatment regiment. EF or SSe when administered singly or in combination had no significant effect on hind paw swelling.

### Arthrogram Scores

Arthrogram score is a more comprehensive variable that indicates the severity of arthritis. Only MTX and its combinations were associated with significant decrease of arthrogram scores (Table II). Similarly to hind paw swelling, reductions in arthrogram scores were more pronounced for the groups treated with combinations of MTX with EF (MTX + EF, MTX + EF + SSe).

TABLE I The effect of preventive treatment with methotrexate (MTX), *E. faecium* (EF), selenium (SSe) and their combinations on hind paws swelling (ml) in AA rats

| Groups of rats | Day 14 | Day 21 | Day 28 |
|---|---|---|---|
| Healthy controls | 1.22 ± 0.02 | 1.25 ± 0.08 | 1.35 ± 0.08 |
| AA controls | 2.20 ± 0.23 | 2.31 ± 0.17 | 2.18 ± 0.22 |
| AA treated with: | | | |
| EF | 2.11 ± 0.44 | 2.20 ± 0.37 | 2.09 ± 0.28 |
| SSe | 2.09 ± 0.26 | 2.20 ± 0.24 | 2.12 ± 0.27 |
| EF + SSe | 2.15 ± 0.19 | 2.36 ± 0.21 | 2.19 ± 0.25 |
| MTX | 1.79 ± 0.27** | 2.01 ± 0.26* | 1.85 ± 0.23** |
| MTX + EF | 1.48 ± 0.22***† | 1.69 ± 0.33***† | 1.58 ± 0.22***† |
| MTX + SSe | 1.81 ± 0.31** | 1.98 ± 0.38* | 1.79 ± 0.36* |
| MTX + EF + SSe | 1.39 ± 0.23***† | 1.65 ± 0.41***† | 1.56 ± 0.27***† |

Data represent mean values ± SD for 10 rats. Significantly different from arthritic control rats: *$p < 0.05$, **$p < 0.01$, ***$p < 0.001$. Significantly different from arthritic rats treated with MTX alone: †$p < 0.05$.

TABLE II The effect of preventive treatment with methotrexate (MTX), *E. faecium* (EF), selenium (SSe) and their combinations on the arthrogram score in rats with AA

| Groups of rats | Day 14 | Day 21 | Day 28 |
|---|---|---|---|
| AA controls | 16.67 ± 1.87 | 19.13 ± 1.83 | 19.33 ± 1.87 |
| AA treated with: | | | |
| EF | 14.63 ± 5.15 | 17.13 ± 2.95 | 18.25 ± 3.20 |
| SSe | 15.00 ± 2.78 | 18.38 ± 2.20 | 19.38 ± 2.72 |
| EF + SSe | 16.25 ± 1.91 | 17.38 ± 1.50 | 19.75 ± 2.43 |
| MTX | 10.50 ± 2.62*** | 14.88 ± 3.31** | 14.00 ± 4.57** |
| MTX + EF | 7.50 ± 1.20***† | 13.50 ± 3.42*** | 12.38 ± 4.37*** |
| MTX + SSe | 10.13 ± 3.40*** | 15.75 ± 2.31** | 14.88 ± 4.73** |
| MTX + EF + SSe | 7.30 ± 2.06***† | 11.00 ± 2.40***† | 11.25 ± 3.08*** |

Data represent mean values ± SD for 10 rats. Significantly different from arthritic control rats: $^{**}p < 0.01$, $^{***}p < 0.001$. Significantly different from arthritic rats treated with MTX alone: $^{\dagger}p < 0.05$.

### Serum Albumin Concentrations

Serum albumin behaves as a negative acute phase reactant in both rat as well as human arthritis. Lower levels of serum albumin corresponded to higher levels of inflammatory activity. The concentration of albumin in the serum of arthritic controls was significantly lower than in healthy controls (HC vs AA $p < 0.001$). All treatments containing MTX significantly inhibited the serum albumin decrease (Table III). EF potentiated the beneficial effect of MTX (on day 28). EF or SSe used singly and their combination EF + SSe without MTX did not influence this inflammatory marker.

### Serum Nitrite/nitrate Concentrations

Serum concentrations of nitrite/nitrate reflect nitric oxide (NO) production in various tissues and inflammatory responses. The clinical onset of AA was associated with significant rise in nitrite/nitrate concentrations. MTX alone and its combinations with EF and SSe significantly decreased nitrite/nitrate concentrations during the whole study compared to arthritic controls (Table IV). SSe, EF or their combination EF + SSe had no significant effect on the serum nitrite/nitrate concentrations, but EF potentiated the beneficial effect of MTX on day 28 of the study.

### Whole Body BMD

Development of arthritis in both humans and rats is associated with the development of osteopenia due to the activation of pro-inflammatory cytokines. At the end of the study (on day 50), AA rats had markedly lower values of the whole body BMD in comparison with healthy controls (Fig. 1). Evaluation of different treatments showed that only the combinations MTX + EF and MTX + EF + SSe inhibited significantly the whole body BMD decrease in AA rats.

### Evaluation of Bone Erosions

X-ray scans of AA rats on day 50 revealed destruction of joint structures characteristic for arthritis. The radiographic scores positively correlated with hind paw swellings; bone erosions increased with increased swelling. No changes were detected in healthy controls, while arthritis was manifested by pronounced destructions (Fig. 2). MTX and its combinations MTX + EF, MTX + SSe significantly reduced radiographic scores. The most pronounced reduction was observed for 3-agent combination MTX + EF + SSe. EF or SSe administered singly or in combination did not influence this parameter.

### DISCUSSION

The purpose of our study was to evaluate the effect of the probiotic agent EF and selenium on MTX treatment of AA in rats. Since the effects on BMD and radiographic scores were also investigated, the duration of the treatment was longer (50 days) than usually administered for AA

TABLE III The effect of preventive treatment with methotrexate (MTX), *E. faecium* (EF), selenium (SSe) and their combinations on serum albumin concentrations (g/L) in rats with AA

| Groups of rats | Day 14 | Day 21 | Day 28 |
|---|---|---|---|
| Healthy controls | 33.18 ± 1.02 | 34.55 ± 1.86 | 34.95 ± 1.84 |
| AA controls | 25.90 ± 0.99 | 26.34 ± 1.21 | 26.40 ± 1.95 |
| AA treated with: | | | |
| EF | 26.33 ± 1.37 | 26.58 ± 0.85 | 25.66 ± 1.79 |
| SSe | 25.61 ± 1.85 | 26.26 ± 0.82 | 24.91 ± 1.47 |
| EF + SSe | 26.04 ± 0.97 | 26.34 ± 1.54 | 25.40 ± 1.66 |
| MTX | 27.15 ± 1.10* | 29.06 ± 2.78* | 28.80 ± 2.11* |
| MTX + EF | 27.81 ± 2.10* | 29.14 ± 2.89* | 29.21 ± 1.24** |
| MTX + SSe | 27.34 ± 1.56* | 28.96 ± 2.74* | 28.84 ± 1.48* |
| MTX + EF + SSe | 28.02 ± 2.62* | 30.25 ± 3.95* | 29.87 ± 2.00** |

Data represent mean values ± SD for 10 rats. Significantly different from arthritic control rats: $^{*}p < 0.05$, $^{**}p < 0.01$.

TABLE IV The effect of preventive treatment with methotrexate (MTX), *E. faecium* (EF), selenium (SSe) and their combinations on serum nitrite/nitrate concentrations (nmol/ml) in rats with AA

| Groups of rats | Day 14 | Day 21 | Day 28 |
|---|---|---|---|
| Healthy controls | 39.75 ± 4.83 | 38.76 ± 8.57 | 40.94 ± 4.71 |
| AA controls | 91.14 ± 14.63 | 84.24 ± 14.70 | 74.69 ± 7.93 |
| AA treated with: | | | |
| EF | 88.21 ± 12.67 | 79.53 ± 10.22 | 71.62 ± 10.69 |
| SSe | 97.79 ± 14.56 | 77.89 ± 8.11 | 78.56 ± 18.87 |
| EF + SSe | 89.72 ± 15.61 | 77.11 ± 16.80 | 70.65 ± 13.09 |
| MTX | 76.82 ± 12.08* | 71.34 ± 8.40* | 67.53 ± 5.51* |
| MTX + EF | 75.51 ± 8.14* | 69.61 ± 7.15* | 58.85 ± 10.97** |
| MTX + SSe | 75.81 ± 11.54* | 69.77 ± 11.64* | 64.51 ± 9.95* |
| MTX + EF + SSe | 74.17 ± 11.74* | 65.77 ± 13.69* | 57.97 ± 13.16** |

Data represent mean values ± SD for 10 rats. Significantly different from arthritic control rats: $^*p < 0.05$, $^{**}p < 0.01$.

(10–14 days) to prevent exacerbation of arthritis after discontinuation of the therapy.

The results of our investigation confirmed the previously reported effect of MTX treatment in rats with AA (Welles *et al.*, 1985). MTX at a dose of 0.6 mg/kg/week suppressed, but did not prevent, arthritis development. In our study, MTX significantly suppressed the hind paw swelling and decreased the arthrogram scores. EF but not SSe potentiated the beneficial effect of MTX, which resulted in a more significant reduction of hind paw swelling and arthrogram scores. Parnham *et al.* (1987) have observed a weak inhibition of carrageenan paw oedema and AA in rats with organo-selenium compound, ebselen.

Serum albumin acts as a negative acute phase reactant in rat arthritis, and the decrease of serum albumin levels reflects the changes in synthesis of this protein in the liver secondary to the activation of hepatic cells by inflammatory cytokines, mainly IL-1 (Connolly *et al.*, 1988). Our results correlate with the observation that MTX markedly prevents the albumin decrease in rat AA (Connolly *et al.*, 1988). The administration of EF was positively manifested in both combinations MTX + EF and MTX + EF + SSe on day 28. The combination of MTX with SSe had no additional effect compared to MTX alone. In patients with RA, the concentration of selenium containing glutathione peroxidase in polymorphonuclear leucocytes is decreased (Tarp, 1994). Supplementation with selenium increases the concentration of selenium in serum and erythrocytes but only weakly increases its concentration in polymorphonuclear leucocytes, and that might be the cause of its insufficient anti-inflammatory effect in arthritis.

NO, unstable free radical produced by the action of the enzyme NO synthase (NOS) on L-arginine, is a mediator of multiple physiologic functions, and may also mediate local inflammation and tissue destruction (Stamler *et al.*, 1992). NO is involved in both initiation and development of AA in rats (Oyanagui, 1994). Moreover, inhibitors of NOS have been shown to suppress arthritis in several animal models (Stefanovic-Racic *et al.*, 1995; Cannon *et al.*, 1996) and increased NO levels have been found in patients with RA (Ueki *et al.*, 1996). Omata *et al.* (1997)

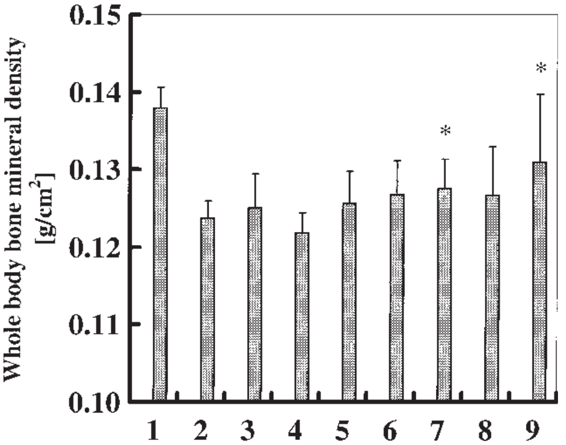

FIGURE 1 The effect of *E. faecium* (EF), selenium (SSe), methotrexate (MTX) and their combinations on whole body bone mineral density (BMD) in rats assessed on day 50. Animals were grouped as follows: (1) nonarthritic untreated controls; (2) AA untreated controls; (3) AA rats treated with EF 15 mg/kg, 5 days a week; (4) AA rats treated with SSe (0.050 mg/kg containing 0.015 mg/kg selenium), 5 days a week; (5) AA rats treated with combination EF + SSe; (6) AA rats treated with MTX 0.6 mg/kg/week; (7) AA rats treated with combination MTX + EF; (8) AA rats treated with combination MTX + SSe; (9) AA rats treated with combination MTX + EF + SSe. Data represent mean values ± SD for 10 rats. Significantly different from arthritic control rats: $^*p < 0.05$.

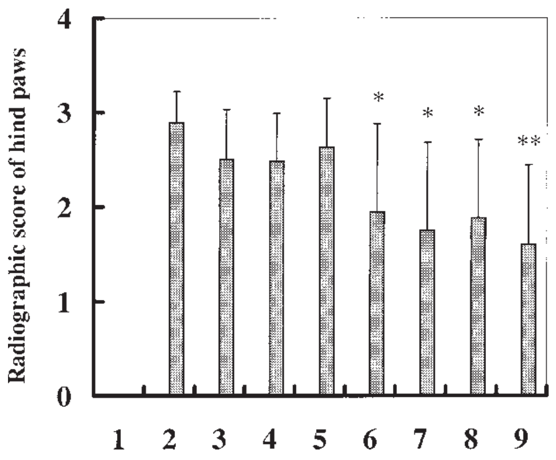

FIGURE 2 The effect of *E. faecium* (EF) selenium (SSe), methotrexate (MTX) and their combinations on radiographic scores in rats assessed on day 50. Designation of rat groups as in Fig. 1. Data represent mean values ± SD for 10 rats. Significantly different from arthritic control rats: $^*p < 0.05$, $^{**}p < 0.01$.

reported MTX to suppress *ex vivo* production of NO in macrophages of rats with AA. In our study, markedly increased serum nitrite/nitrate concentrations were measured in AA rats. EF or SSe administered singly or in combination did not decrease the concentration of nitrite/nitrate but EF potentiated the inhibitory effect of MTX on day 28.

AA rats developed marked osteopenia predominantly in the distal periarticular region of the femur as a result of the suppression of bone formation and increased bone resorption (Bonnet *et al.*, 1993). Suzuki *et al.* (1997) reported short-term low doses of MTX to ameliorate abnormal bone metabolism and decrease the bone loss in AA. In AA rats, MTX in the dose of 3 mg/kg/week suppressed arthritis and restored the decreased osteogenic activity of bone marrow cells, and significantly increased periarticular BMD in the femur. The lower dose of 0.3 mg/kg/week had no effect on femoral BMD. In our study, MTX (0.6 mg/kg/week) had no effect on the whole body BMD in rats. Interestingly EF alone, similarly to MTX, was ineffective. But EF in combination with MTX significantly reduced the whole body BMD loss in AA rats. This observation needs further study and measurements of BMD in different sites (e.g. BMD of femur or tibia).

Bone erosions of hind paws are typical sign of chronic arthritis; reduction of radiographic response in AA with MTX is dose-dependent. Low doses of MTX (0.1 or 0.2 mg) did not normalize the radiographic findings in AA (Kawai *et al.*, 1997). Morgan *et al.* (2001) found that MTX at 1 mg/kg/week resulted in mean total radiographic scores not different from healthy controls; lower or higher doses of MTX were less effective. In our experiment, MTX 0.6 mg/kg/week significantly decreased the radiographic scores. The more pronounced reduction of radiographic scores with 3-agent combination MTX + EF + SSe may be result of additional effect of probiotic agent, SSe or their combination.

Our results demonstrate that administration of probiotic agent EF alone does not worsen the clinical, inflammatory, or destructive markers of AA. On the contrary, it potentiates the beneficial effect of MTX treatment on arthritis-associated inflammation and destruction. Our results also show that the beneficial effect of selenium-enriched EF observed in our previous study (Rovensky *et al.*, 2002) results mainly from the bacteria and not from selenium. The possible explanation may be the altered intestinal absorption of MTX after colonization of gut by this probiotic agent. During the experiment no diarrhea was observed in rats. Mao *et al.* (1996) have shown that administration of lactobacilli, especially *L. plantarum*, is helpful in reducing the severity of the MTX-induced enterocolitis in rats. Due to the fact that EF not only increases the efficacy of MTX treatment but may favourably influence the intestinal flora and may protect against related infectious diseases, we can see rationale for the use of this probiotic agent for the MTX treatment of arthritis.

The focus of the present study is on the long-term preventive treatment of arthritic rats; further studies are needed to explore the role of EF in MTX therapy regimen and the possible therapeutical use of this probiotic agent in arthritis.

### *References*

Belicová, A., Krajčovič, J., Dobias, J. and Ebringer, L. (1999) "Antimutagenicity of milk fermented by *Enterococcus faecium*", *Folia Microbiol.* **44**, 513–518.

Bonnet, J., Zerath, E., Picaud, N., *et al.* (1993) "Bone morphometric changes in adjuvant-induced polyarthritic osteopenia in rats: evidence for an early bone formation defect", *J. Bone Miner. Res.* **8**, 659–668.

Cannon, G.W., Openshaw, S.J., Hibbs, J.B., Jr., Hoidal, J.R., Huecksteadt, T.P. and Griffiths, M.M. (1996) "Nitric oxide production during adjuvant-induced and collagen induced arthritis", *Arthritis Rheum.* **39**, 1677–1684.

Cebra, J.J. (1999) "Influences of microbiota on intestinal immune system development", *Am. J. Clin. Nutr.* **69**, 1046–1051.

Chen, T., Isomäki, P., Rimpiläinen, M. and Toivanen, P. (1999) "Human cytokine responses induced by Gram-positive cell walls of normal intestinal microbiota", *Clin. Exp. Immunol.* **118**, 261–267.

Connolly, K.M., Stecher, V.J., Danis, E., Pruden, D.J. and LaBrie, T. (1988) "Alteration of interleukin-1 production and the acute phase response following medication of adjuvant arthritic rats with cyclosporin-A or methotrexate", *Int. J. Immunopharmacol.* **10**, 717–728.

Cortas, N.K. and Waking, N.W. (1990) "Determination of inorganic nitrate in serum and urine by a kinetic cadmium-reduction method", *Clin. Chem.* **36**, 1440–1443.

Eerola, E., Möttö, T., Hannonen, P., *et al.* (1994) "Intestinal flora in early rheumatoid arthritis", *Br. J. Rheumatol.* **33**, 1030–1038.

Ferenčík, M., Mikeš, Z., Ebringer, L., Jahnová, E. and Čižmár, I. (2000) "Immunostimulatory and other beneficial health effects of lactic acid bacteria", *Bratisl. Lek. Listy* **101**, 51–53.

Hatakka, K., Martio, J., Korpela, M., *et al.* (2003) "Effects of probiotic therapy on the activity and activation of mild rheumatoid arthritis—a pilot study", *Scand. J. Rheumatol.* **32**, 211–215.

Isolauri, E., Sütas, Y., Kankaanpää, P., Arvilommi, H. and Salminen, S. (2001) "Probiotics: effect on immunity", *Am. J. Clin. Nutr.* **73**(Suppl.), 444S–450S.

Kano, H., Kaneko, T. and Kaminogawa, S. (2002) "Oral intake of *Lactobacillus delbrueckii* subsp. *bulgaricus* OLL1073R-1 prevents collagen-induced arthritis in mice", *J. Food Prot.* **65**, 153–160.

Kawai, S., Nagai, K., Nishida, S., Sakio, K., Murai, E. and Mizushima, Y. (1997) "Low-dose pulse methotrexate inhibits articular destruction of adjuvant arthritis in rats", *J. Pharm. Pharmacol.* **49**, 213–215.

Malin, M., Verronen, P. and Korhonen, A. (1997) "Dietary therapy with *Lactobacillus GG*, bovine colostrum or bovine immune colostrum in patients with juvenile chronic arthritis: evaluation of effect on gut defense mechanism", *Inflammopharmacology* **5**, 219–236.

Mao, Y., Nobaek, S., Kasravi, B., *et al.* (1996) "The effect of *Lactobacillus* strains and oat fiber on methotrexate-induced enterocolitis in rats", *Gastroenterology* **111**, 334–344.

McDougall, J., Bruce, B., Spiller, G., Westerdahl, J. and McDougall, M. (2002) "Effects of a very low fat, vegan diet in subjects with rheumatoid arthritis", *J. Altern. Complement. Med.* **8**, 71–75.

Mikeš, Z., Ferenčík, M., Jahnová, E., Ebringer, L. and Čižnár, I. (1995) "Hypocholesteloremic and immunostimulatory effects of orally applied *Enterococcus faecium* M-74 in man", *Folia Microbiol.* **40**, 639–646.

Morgan, S.L., Baggott, J.E., Bernreuter, W.K., Gay, R.E., Arani, R. and Alarcón, G.S. (2001) "MTX affects inflammation and tissue destruction differently in the rat AA model", *J. Rheumatol.* **28**, 1476–1481.

Omata, T., Segawa, Y., Inoue, N., Tsuzuike, N., Itokazu, Y. and Tamaki, H. (1997) "Methotrexate suppresses nitric oxide production *ex vivo* in macrophages from rats with adjuvant-induced arthritis", *Res. Exp. Med.* **197**, 81–90.

Oyanagui, Y. (1994) "Nitric oxide and superoxide radical are involved in both initiation and development of adjuvant arthritis in rats", *Life Sci.* **54**, PL285–PL289.

Parnham, M.J., Winkelmann, J. and Leyck, S. (1983) "Macrophage, lymphocyte and chronic inflammatory responses in selenium deficient rodents. Association with decreased glutathione peroxidase activity", *Int. J. Immunopharmacol.* **5**, 455–461.

Parnham, M.J., Leyck, S., Kuhl, P., Schalkwijk, J. and van der Berg, W.B. (1987) "Ebselen a new approach to the inhibition of peroxide-dependent inflammation", *Int. J. Tissue React.* **9**, 45–50.

Peltonen, R., Nenonen, M., Helve, T., Hanninen, O., Toivanen, P. and Eerola, E. (1997) "Faecal microbial flora and disease activity in rheumatoid arthritis during a vegan diet", *Br. J. Rheumatol.* **36**, 64–68.

Peretz, A., Siderova, V. and Néve, J. (2001) "Selenium supplementation in rheumatoid arthritis investigated in a double blind, placebo controlled trial", *Scand. J. Rheumatol.* **30**, 208–212.

Rayman, M.P. (2000) "The importance of selenium to human health", *Lancet* **356**, 233–241.

Reid, G., Jass, J., Sebulsky, M.T. and McCormick, J.K. (2003) "Potential uses of probiotics in clinical practice", *Clin. Microbiol. Rev.* **16**, 658–672.

Rovensky, J., Svik, K., Stancikova, M., Istok, R., Ebringer, L. and Ferencik, M. (2002) "Treatment of experimental adjuvant arthritis with the combination of methotrexate and lyophilized *Enterococcus faecium* enriched with organic selenium", *Folia Microbiol.* **47**, 573–578.

Sartor, R.B. (1997) "Review article: Role of enteric microflora in the pathogenesis of intestinal inflammation and arthritis", *Aliment Pharmacol. Ther.* **11**, 17–32.

Simelyte, E., Rimpiläinen, M., Lehtonen, I., Zhang, X. and Toivanen, P. (2000) "Bacterial cell wall-induced arthritis chemical composition and tissue distribution of four *Lactobacillus* strains", *Infect. Immun.* **68**, 3535–3540.

Stamler, J.S., Singel, D.J. and Loscalzo, J. (1992) "Biochemistry of nitric oxide and its redox-activated forms", *Science* **258**, 1898–1902.

Stefanovic-Racic, M., Meyers, K., Meschter, C., Coffey, J.W., Hoffman, R.A. and Evans, C.H. (1995) "Comparison of the nitric oxide synthase inhibitors methylarginine and aminoguanidine as prophylactic and therapeutic agents in rat adjuvant arthritis", *J. Rheumatol.* **22**, 1992–1998.

Suzuki, Y., Nakagawa, M., Masuda, C., *et al.* (1997) "Short-term low dose methotrexate ameliorates abnormal bone metabolism and bone loss in adjuvant-induced arthritis", *J. Rheumatol.* **24**, 1890–1895.

Tarp, U. (1994) "Selenium and selenium-dependent gluthatione peroxidase in rheumatoid arthritis", *Dan. Med. Bull.* **41**, 264–274.

Tlaskalová-Hogenová, H., Stepanková, R., Hudcovic, T., *et al.* (2004) "Commensal bacteria (normal microflora), mucosal immunity and chronic inflammatory and autoimmune diseases", *Immunol. Lett.* **93**, 97–107.

Ueki, Y., Miyake, S., Tominaga, Y. and Eguchi, K. (1996) "Increased nitric oxide levels in patients with rheumatoid arthritis", *J. Rheumatol.* **23**, 230–236.

Welles, W.L., Silkworth, J., Oronsky, A.L., Kerwar, S.S. and Galivan, J. (1985) "Studies on the effect of low dose methotrexate in adjuvant arthritis", *J. Rheumatol.* **12**, 904–906.

